# The influence of a Sprint optimization and training intervention on time spent in the electronic health record (EHR)

**DOI:** 10.1093/jamiaopen/ooab073

**Published:** 2021-08-23

**Authors:** Amber Sieja, Melanie D Whittington, Vanessa Paul Patterson, Katie Markley, Heather Holmstrom, Stephen Rotholz, Christine Gonzalez, Michael Scott Carpenter, Chen-Tan Lin

**Affiliations:** 1 Department of Medicine, University of Colorado School of Medicine, Aurora, Colorado, USA; 2 Navigation Lab, Data Science to Patient Value, University of Colorado School of Medicine, Aurora, Colorado, USA; 3 University of Colorado Health Medical Group and University of Colorado Health, Aurora, Colorado, USA; 4 Department of Family Medicine, University of Colorado School of Medicine, Aurora, Colorado, USA; 5 Department of Obstetrics and Gynecology, University of Colorado School of Medicine, Aurora, Colorado, USA; 6 University of Colorado Health, Aurora, Colorado, USA

**Keywords:** burnout, electronic medical record, electronic health record, training, optimization

## Abstract

**Objective:**

We report the influence of Sprint electronic health record (EHR) training and optimization on clinician time spent in the EHR.

**Materials and Methods:**

We studied the Sprint process in one academic internal medicine practice with 26 providers. Program offerings included individualized training sessions, and the ability to clean up, fix, or build new EHR tools during the 2-week intervention. EHR usage log data were available for 24 clinicians, and the average clinical full-time equivalent was 0.44. We used a quasi-experimental study design with an interrupted time series specification, with 8 months of pre- and 12 months of post-intervention data to evaluate clinician time spent in the EHR.

**Results:**

We discovered a greater than 6 h per day reduction in clinician time spent in the EHR at the clinic level. At the individual clinician level, we demonstrated a time savings of 20 min per clinician per day among those who attended at least 2 training sessions.

**Discussion:**

We can promote EHR time savings for clinicians who engage in robust EHR training and optimization programs. To date, programs have shown a positive correlation between participation and subjective EHR satisfaction, efficiency, or time saved. The impact of EHR training and optimization on objective time savings remains elusive. By measuring time in the EHR, this study contributes to an ongoing conversation about the resources and programs needed to decrease clinician EHR time.

**Conclusions:**

We have demonstrated that Sprint is associated with time savings for clinicians for up to 6 months. We suggest that an investment in EHR optimization and training can pay dividends in clinician time saved.

## INTRODUCTION

Electronic health record (EHR) burden and its relationship to clinician burnout has been well-described,^1–5^ yet 86% of office-based practices use EHRs.^1^ In one study, 70% of clinicians reported health information technology-related stress.^2^ It is common for clinicians to attribute administrative burden to the EHR.^4^ Clinicians note that they spend as much or more time on clerical tasks than they spend in face-to-face interactions with patients and this has been quantified.^6–10^ Measured by system usability scores, EHRs were given an “F” by clinicians, and there was a strong dose–response between usability and provider burnout.^1^ On the contrary, a study of 72,000 EHR users in a national EHR research collaborative suggested that less than 20% of all variation in EHR experience was due to the particular EHR platform and 50% of variation occurred at the physician user level.^11^ Thus, effective EHR personalization, training, and optimization may be the key to improving EHR satisfaction and usability.^4^^,^^11–16^ Realizing the prevalence of disillusionment in clinicians, we need to understand how to engage this population in EHR improvement efforts.^17^^,^^18^

Previously, we reported that Sprint optimization events increase EHR satisfaction, improve teamwork, and reduce burnout.^12^ Other studies reveal user-reported decreases in EHR time after training and optimization interventions.^4^^,^^13^ One program used a home-grown objective metric to evaluate EHR time.^19^ In this article, we provide an updated description of our Sprint EHR training and optimization program and demonstrate objective clinician EHR time savings post-intervention.

## OBJECTIVE

We report the influence of Sprint EHR training and optimization on clinician time spent in the EHR.

## MATERIALS AND METHODS

In July 2018, one University of Colorado clinic comprised of 23 internists and 3 advanced practice providers participated, without financial incentive, in a 2-week Sprint EHR training and optimization event (Table 1).^12^ This clinic uses the Epic EHR (Epic Systems, Verona, WI, USA). Sprint is a 2-week, on site, clinic-specific EHR optimization and training event conducted by an 11-member multidisciplinary team. Sprint employs Agile methodology, rapid build cycles and iterations of EHR tools and clinical workflows.

**Table 1.** Demographics of Sprint clinician participants

	Gender			Clinical FTE (cFTE)^a^		
	Female	Male	Other	<0.3	0.3–0.59	0.6–1
Number of APPs	2	0	0	1	1	0
Number of physicians	16	7	1	10	8	6

cFTE, clinical FTE (0.1 cFTE = 4-h clinic session).

All clinicians and hospital-employed clinical staff are scheduled for 2 90 min one-to-one training sessions approximately 90 days prior to Sprint. Sessions can be canceled, changed, or added as requested. Ambulatory-certified EHR trainers conduct individual training sessions in the clinic, initiating their sessions with workflow inquiry and including time to address end user questions. Then, role-specific core competency training checklists are employed to provide consistent content when teaching the resultant high-efficiency workflows. All checklists are divided into 4 core areas of EHR work: documentation, in basket, clinical review, and ordering (Supplementary Appendix A). Speech-recognition training and one physician informaticist-directed group training session, focused on EHR personalization, are also offered. All training was performed using a combination of auditory, visual, and kinesthetic approaches tailored to the needs and preferences of the individual trainee.

While the Sprint trainers educated clinicians and staff, the Sprint EHR ambulatory analyst team helped to address 69 internal medicine clinician and staff requests to remediating broken, outdated, missing EHR tools during the Sprint event. Several of these requests were addressed at the user level with training while others required analysts to “clean-up” existing build (removing departed providers and/or updating patient portal message routing for newer providers). The largest net new build impacts for this clinic were the development of a comprehensive order preference list and a change from workstation printing to print profiles. The order preference list and print profiles provided the clinician with local control and fewer choices when placing orders and when printing orders, visit summaries and letters. Other build changes included: (1) a new 6-min walk oxygen titration flowsheet, (2) new quick links for populating the visit note with recent common lab values (thyroid, lipids, inr), (3) updates to existing disease-specific reports, and (4) updates to ancillary role templates to provide access to missing tools.

We analyzed Sprint clinician EHR usage data from December 2017 (earliest data available, 8 months pre-intervention) through July 2019 (12 months post-intervention). Data were retrieved from Epic Systems Provider Efficiency Profile (PEP) (December 2017–November 2018) and Epic Signal reports (December 2018–July 2019). Study investigators held key informant interviews with health system informaticists to confirm comparability in the outcomes selected for our analysis between the 2 reporting platforms. The time outcome definitions and data capture were consistent between the 2 platforms, and thus the outcomes evaluated included average daily clinician time spent in the EHR stratified by time spent in in-basket, orders, notes, and clinical review areas. Other outcomes were not able to be evaluated due to potential differences in how these data elements were defined and captured by the 2 different reporting systems. De-identified data are available upon reasonable request.

The data were analyzed at the clinician level as average daily values over each month, based on monthly clinical EHR activity and the number of working days in the month; the unit of analysis was the average clinician-working day. The mean and 95% confidence interval (CI) of each outcome was calculated for the pre- and post-intervention periods. Multivariate ordinary least squares (OLS) regression was used to identify the association of Sprint with the average time spent in the EHR per clinician per day. The primary dependent variable (*Sprint*) was an interaction variable between the period after the implementation of Sprint (*Post*) and the number of training sessions attended by the clinician (*Training*). The model also controlled for full-time equivalent (*FTE*) and the average patient age (*Age*) to account for variation in the workforce and patient severity. To account for repeated measures, the standard errors were clustered at the clinician level.

Data were then aggregated and analyzed at the unit level using an interrupted time series specification to account for trends over time and to identify the duration of benefits from the intervention. An interrupted time series specification is designed to assess the effects of an independent variable (the occurrence of the Sprint) on a repeated measure of the process of interest (in our analysis the time spent in the EHR). Researchers have advocated for the greater use of interrupted time series analyses in the evaluation of intervention research due to its ability to account for trends over time, simulate an internal comparison group, and be a rigorous approach when a randomized controlled trial may not have been feasible or had not be done. A characteristic of time series data is the dependence between consecutive data points. Thus, Prais-Winsten generalized least squares regression was used to estimate the change in outcomes following the Sprint intervention, while controlling for clinic FTE. With standard errors following a first-order autoregressive process, as is characteristic with repeated measures inherent to the time series data, Prais-Winsten estimation accounts for the serial correlation in the error term while estimating the parameters in a linear model.

## RESULTS

Of 26 clinicians in this internal medicine practice, 24 (93%) of them, 22 internists and 2 advanced practice providers, had available data throughout the pre- and post-intervention time that could be used in our analysis. The average clinical FTE for the 24 clinicians studied was 0.44 at the time of the Sprint event. All clinicians in our study completed at least a single one-on-one training session during Sprint and 14 (58%) clinicians completed 2 sessions.

Before Sprint, a provider spent an average of 88.4 (95% CI: 81.06, 95.76) min per day in the EHR, with 16.96 (95% CI: 15.61, 18.30) min per day in in-basket, 21.81 (95% CI: 19.61, 24.01) min per day in orders, 31.28 (95% CI: 28.35, 34.21) min per day in notes and letters, and 18.36 (95% CI: 16.65, 20.07) min per day in clinical review. After Sprint, a provider spent an average of 76.26 (95% CI: 71.59, 80.93) min per day in the EHR, with 16.42 (95% CI: 15.44, 17.41) min per day in in-basket, 14.02 (95% CI: 12.97, 15.06) min per day in orders, 28.83 (95% CI: 26.81, 30.85) min per day in notes and letters, and 16.99 (95% CI: 15.83, 18.16) min per day in clinical review.

Table 2 presents the unadjusted average time spent by an individual clinician in each of the 4 EHR work areas examined, before and after Sprint.

**Table 2.** Unadjusted clinician time spent (minutes per day) in EHR work areas pre- and post-Sprint (mean, 95% CI)

	In-basket	Orders	Notes/letters	Clinical review	Total time
Pre-Sprint	16.96 (15.61, 18.30)	21.81 (19.61, 24.01)	31.28 (28.35, 34.21)	18.36 (16.65, 20.07)	88.4 (81.06, 95.76)
Post-Sprint	16.42 (15.44, 17.41)	14.02 (12.97, 15.06)	28.83 (26.81, 30.85)	16.99 (15.83, 18.16)	76.26 (71.59, 80.93)

Table 3 presents the results from the multivariate OLS regression at the clinician level. The values in Table 3 represent the coefficients on each parameters from the OLS regression. Individuals that attended one training session only experienced significant time savings in the orders bucket; however, no reductions in time spent was observed when looking across all buckets for individuals that only attended on training session. Attending 2 Sprint training sessions was associated with a significant reduction in time spent in the EHR of approximately 20 min per clinician per day. Significant reductions in time were not observed for clinicians who only attended one training session. As expected, a provider with more clinical FTE spent more time in the EHR.

**Table 3.** Association of Sprint with average clinician time (minutes per day) spent in the EHR

	In-basket	Orders	Notes/letters	Clinical review	Total time
Sprint					
1 training session	2.97 (1.59)	−4.9 (1.60)^a^	4.13 (3.75)	3.50 (1.81)	5.70 (7.05)
2 training sessions	−2.19 (0.92)^a^	−8.55 (1.51)^a^	−5.70 (2.61)^a^	−3.70 (1.49)^a^	−20.14 (5.12)^a^
FTE	26.56 (3.19)^a^	31.44 (7.32)^a^	39.41 (11.55)^a^	26.78 (6.19)^a^	124.19 (25.11)^a^
Age	0.15 (0.08)	−0.10 (0.13)	−0.76 (0.25)^a^	−0.16 (0.20)	−0.86 (0.57)
Constant	−3.84 (4.95)	14.07 (7.97)	59.48 (15.20)^a^	16.24 (12.46)	85.95 (35.01)^a^

Regression coefficient with robust standard error in parentheses.

Significant at *P *<* *0.05.

**Figure 1.**
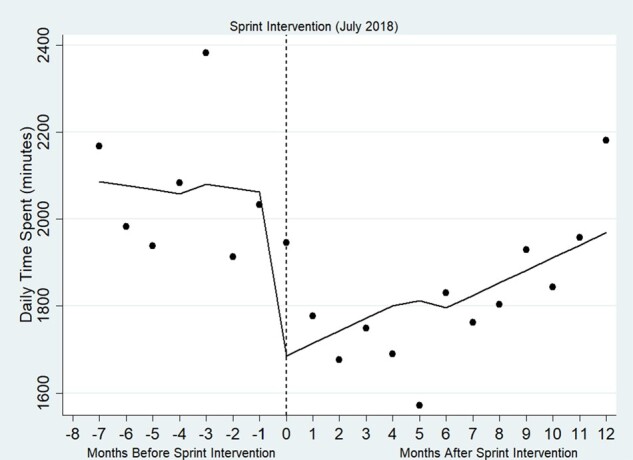
Average daily EHR time spent by the clinic pre- and post-Sprint intervention.

Figure 1 presents results of the interrupted time series specification and reveals the daily time spent in the EHR at the clinic level before and after Sprint. Prior to the Sprint intervention, the clinic was spending approximately 2000 min per day in the EHR. The interrupted time series analysis provides 3 numbers: the trend observed prior to the Sprint intervention, the immediate impact of the Sprint intervention, and the trend observed following the Sprint interventions. For the trend observed prior to the Sprint intervention, the interrupted time series estimated a negative, although insignificant trend of −9.14 min (95% CI: −59.06, 40.78) prior to Sprint. Therefore, prior to Sprint, time in the EHR was relatively constant but trending toward reductions in time per day. For the immediate impact of the Sprint intervention observed immediately after Sprint implementation, there was an immediate and significant reduction in time spent in the EHR of −367.87 (95% CI: −32.22, −703.52) min per day. For the trend observed prior to the Sprint intervention, there was no significant ongoing reduction in time following the Sprint. This was showcased by a positive, but insignificant value in the trend in the post-period of 38.02 min (95% CI: −52.23, 128.27). These results from the interrupted time series analysis suggest an immediate, one-time time savings, but limited ongoing time savings. As depicted in Figure 1, 6 months following the Sprint intervention, the daily time in EHR started to increase. Twelve months following the intervention, daily time spent in the EHR was at similar levels compared with the period prior to the Sprint intervention.

## DISCUSSION

An intensive, on-site Sprint EHR optimization event in one internal medicine practice was associated with a greater than 6 h per day clinic-level EHR time savings that appeared to be sustained over 6 months. Time spent in the EHR returned approximately to pre-intervention levels within 12 months of the Sprint intervention suggesting that annual Sprint refreshers may be helpful, which is concordant with the recommendations of groups who focus on training to increase EHR satisfaction and efficiency.^11^

We would expect that EHR upgrades would enhance workflows and improve efficiency, but it is possible that clinicians, after only one Sprint intervention, lack sufficient training to discover and optimize new tools and workflows. Our institution had 4 EHR upgrades (on 11 August 2018, 26 January 2019, 6 April 2019, and 20 July 2019) that overlapped with our post-Sprint intervention analysis time period. This quarterly upgrade cadence is common in large organizations who rely heavily on vendor-development to improve local EHR user experience. Quarterly upgrades generally allow for smaller, more incremental change in the EHR system. In contrast, we had significant changes to ordering workflows with our August 2018 upgrade. At our organization, each EHR upgrade is prefaced by a system-wide optimization newsletter without required training or at-the-elbow support. It is plausible that software upgrades impacted our clinic-level study data which reveal a return to baseline in clinician EHR time 6–12 months after Sprint. In fact, we suspect that our study participants returned to more familiar, less efficient workflows when EHR changes occurred. Although not studied, it is also possible that turnover of clinical staff could affect clinical workflow and EHR time. In this study, Sprint was the first dedicated training intervention for this internal medicine clinic since implementing the EPIC EHR 7 years earlier. As a result, we observed information overload for some clinicians which could also contribute to a shortened interval for sustained workflow efficiencies. As our program has moved into the second round of Sprints for many clinics, we have been able to focus on solidifying and maintaining basic EHR knowledge which is in stark contrast to “first round” Sprints where the majority of training content was net new for clinicians.

Sprint is designed to improve EHR confidence and teamwork, which we anticipate will grow incrementally and be additive with each additional Sprint event. Sprint highlights the role of the EHR in key workflows and encourages care team members to problem-solve using the EHR as a tool rather than perceiving it as a barrier. Sprint highlights clinic personnel who are EHR experts and allows them to serve as local resources between Sprints. These “EHR champions” could be used by organizations to digest quarterly EHR optimization bulletins, highlighting and redistributing communication on upgrade items that impact their clinic. It is possible that mandatory quarterly optimization training modules would also help with efficiency losses resulting from EHR upgrades, but this is likely to be unpopular with clinicians. Primarily due to organizational size and Sprint cost, our goal is to conduct Sprint events in each clinic every 2 years to minimize time without dedicated at-the-elbow training. We believe our study is the first to report a significant decrease in EHR time based on data from EHR usage logs analyzed over a 1-year period following an intervention. We found that clinicians participating in 1 90-min individual training did not demonstrate time savings after Sprint. However, clinicians participating in 2 90-min individual trainings realized an average time savings of 20 min per day. To ensure career success, academic faculty and other health care providers require training in areas that include procedures and devices, billing and coding accuracy, and continuing medical education. However, we lack standards^14^ and typically do not require ongoing training in optimal usage of the EHR, the tool with which clinicians spend most of their time. A national EHR research collaborative suggested that 3–5 h of EHR training per year “sets clinicians up for success” and that every hour of training increases client satisfaction with their EHR experience.^11^ Similarly, we found that 3 h of individualized training with Sprint was associated with a 20 min per day reduction in clinician time.

Many authors have suggested we must understand the human experience in EHR use, and they have reminded us that different learners view the same EHR interface differently.^15^^,^^19^^,^^20^ In our experience, this creates a challenge for designing a training curriculum that can be distributed broadly via white papers or videos. Investing in clinicians and staff to meet them where they are as learners, in their physical spaces^12^^,^^19^^,^^20^ or within their specialty areas, can be very effective. Adhering to these principles, the Sprint team physically inserts themselves for 2 weeks, full-time into ambulatory clinics to observe, listen to, translate and partner with clinicians and staff. To promote widespread engagement, we slow down, increase repetition or schedule additional sessions for some trainees, while we increase cadence and inspire advanced customization when desired for others.

According to a recent report by the American Medical Association, almost 50% of primary care clinicians report burnout.^21^ Physician burnout has been linked to decreased patient satisfaction and lower quality of care.^22–24^ KLAS Net Experience Score data reveal that “the more satisfied providers are…with their EHR, the less likely it is that many providers in that organization are experiencing burnout.”^11^ Sprint team members empathize with clinicians, but they also empower them to see the EHR not as a burden but as a source of information to aide in clinical decision-making and patient engagement. Similarly, clinical staff learn how to support clinicians and patients not by adding work but by doing work more efficiently and confidently. Sprint has removed key barriers to EHR optimization and employed facilitators including dedicated resources, connection with users, informaticist leaders, and strong engagement.^17^^,^^18^^,^^25^^,^^26^

We agree that we need to encourage our colleagues to “take advantage of the technology without slavishly replicating old paper-based workflows.”^27^ This is a tangible goal, but it will require the engagement of multidisciplinary clinical teams who possess the clinical and the EHR knowledge needed to facilitate meaningful change in their practices. While further study and repeated measurements will be needed to determine if clinician time savings and EHR satisfaction is sustainable with ongoing and repeated Sprint events, our organization plans to continue to support Sprint teams as an effective mechanism to engage and improve EHR training and optimization in our ambulatory practices.

### Limitations

In this study, we are limited by our inability to separate out the contributions of tool optimization and training to EHR time savings. The tools most likely to help decrease EHR time in this Sprint included customizations that can be built by end users, trainers or analysts. In fact, the order preference list, perhaps our most impactful “build” for this Sprint was designed by clinical end users with physician informaticist guidance, but it was aggressively trained as part of Sprint core competencies. In Sprints, we have discovered that most new functionality and substantially altered build requires training. Thus, an analysis to separate out the contributions of each would be near impossible. The generalizability of our results to other clinical settings is limited because we relied on a single EHR and studied the impact of Sprint in only one ambulatory clinic. Our usage logs originated with our EHR vendor, but we used equivalent data sources pre- and post-intervention. We did not survey clinicians on EHR satisfaction longitudinally after Sprint, so we cannot determine whether EHR satisfaction waned with time or with the EHR upgrades. The clinic in this study was the first to receive Sprint core competency training, so it is possible that there was some variability in how information was conveyed with our new training process. Although data on time spent in the EHR by our 21 staff participants is not available, it should be noted that staff participated in 2 90-min, role-specific training sessions during this Sprint. While we updated and changed clinic workflows during Sprint, we did not redistribute buckets of work from clinicians to staff that could have impacted time spent in the EHR post-Sprint. Our staff-to-provider clinical FTE ratios remained consistent throughout the study. Finally, we had limited data available to account for patient severity in our analyses. The best proxy we could identify was patient age, but we acknowledge that this is a weak proxy and should be interpreted as such.

## CONCLUSION

The diagnosis and treatment of physician burnout and wellness remains a challenge. We have demonstrated that Sprint is associated with time savings for clinicians for up to 6 months.

## DATA AVAILABILITY

De-identified data are available upon reasonable request.

## AUTHOR CONTRIBUTIONS

AS led this Sprint event and was the primary author for the Background, Methods and Discussion section of this manuscript. KM, HH, and SR lead the design and implementation of the Sprint training and optimization program and contributed to manuscript revisions. VPP and MDW independently procured user log data and analyzed and constructed the Data and Results sections of this manuscript. CG and MSC lead and innovate within the Sprint program and they drove collection of provider and training metrics. C-TL sponsors Sprint work and participated in framing and actively reviewing and editing this manuscript.

## SUPPLEMENTARY MATERIAL

Supplementary material is available at *Journal of the American Medical Informatics Association* online.

## Supplementary Material

ooab073_Supplementary_Data
