# Supplementary material for: The influence of a Sprint optimization and training intervention on time spent in the electronic health record (EHR)
Source: JAMIA Open. 2021 Aug 23;4(3):ooab073. doi: 10.1093/jamiaopen/ooab073 (PMC8382277; doi:10.1093/jamiaopen/ooab073)
Supplement: ooab073_Supplementary_Data [file ooab073_supplementary_data.docx]

| **Core Competencies** | **Competency Definition** | **Date/ Trainer** |
| --- | --- | --- |
| **Chart Review (7)** |  |  |
| Chart Search | Find patient-specific information quickly |  |
| Chart Filters | Highlight relevant information quickly |  |
| Results Review | Review lab trends versus individual orders |  |
| Synopsis Reports | Patient and disease specific reports |  |
| Find Result Notes/Result Letters | Retrieve clinicians’ past advice |  |
| My Sticky Note/My Specialty Note | Patient-specific, off-the-record, personal and clinician team notes |  |
| Encounter Tab Benefits | Quickly addend and review past encounter notes/data |  |
| **In Basket (9)** |  |  |
| Build/Add My List | Send in basket note quickly/accurately |  |
| Quick Note | General quick communication |  |
| Result Note | Results to patient/team quick communication |  |
| Result Letter | Results to patient/referrer quick communication |  |
| Postpone | Avoid using in basket as file folder |  |
| Provide Coverage | How to support colleagues and take time off |  |
| Tickler File | Help patients by setting reminder to care team to order/retrieve critical follow up |  |
| Share Info/Results With Another Provider | Quickly inform care team of treatment plan changes |  |
| Quick Actions | Automate frequently used in basket workflows |  |
| **Documentation (6)** |  |  |
| Smarttools Handout | Consolidated and tested documentation shortcuts deemed most utilized by clinicians |  |
| Offer Dragon | Train/customize speech-recognition software |  |
| Autocorrect Dictionary | Convert individual clinician shorthand to medicolegally accurate documentation |  |
| Smartphrases | Documentation tool that prevents re-typing frequently stated information |  |
| Smartblock Macros | Documentation tool that prevents re-selecting same constellation of physical exam findings |  |
| Custom Note Templates | Customized specialty, subspecialty or individual note templates |  |
| **Ordering (11)** |  |  |
| Create Favorite Orders | Set of commonly place orders with pre-populated, frequently-selected items |  |
| Create Order Panels | Disease or evaluation specific orders clustered into one orderable selection |  |
| Edit Favorites | Understand how to change personalized orders |  |
| Organize Favorites | Understand how to re-organize personalized orders |  |
| Order Composer | Understand how to share personalized orders |  |
| Edit Multiple | Quickly update one order parameter on an entire set of patient orders |  |
| Add on labs | Quickly add orders to previously collected patient specimens |  |
| Up-to-date in Meds and Orders and Problem List | Allow EHR to search clinical guidelines with one click |  |
| Change Dx/Re-associate | Update associated billable or test diagnoses quickly |  |
| Patient-reported Med/Verify Available Strengths | Add medications to list without prescribing; avoid fragmentation of prescription data in record |  |
| Erroneous Encounter | Shortcut to close no-show encounters |  |
